# An update of the tsetse fly (Diptera: Glossinidae) distribution and African animal trypanosomosis prevalence in north-eastern KwaZulu-Natal, South Africa

**DOI:** 10.4102/ojvr.v83i1.1172

**Published:** 2016-06-09

**Authors:** Chantel J. de Beer, Gert J. Venter, Karin Kappmeier Green, Johan Esterhuizen, Daniel G. de Klerk, Jerome Ntshangase, Marc J.B. Vreysen, Ronel Pienaar, Makhosazana Motloang, Lundi Ntantiso, Abdalla A. Latif

**Affiliations:** 1 Agricultural Research Council – Onderstepoort Veterinary Institute, Parasites, Vectors & Vector-borne Diseases, South Africa; 2 Department of Zoology and Entomology, University of the Free State, South Africa; 3 Department of Veterinary and Tropical Diseases, University of Pretoria, South Africa; 4 Department of Vector Biology, Liverpool School of Tropical Medicine, United Kingdom; 5 Joint Food and Agriculture Organization/International Atomic Energy Agency Division of Nuclear Techniques in Food and Agriculture, Insect Pest Control Laboratory, Austria; 6 Makhathini Research Station, Jozini, South Africa; 7 College of Agriculture, Engineering and Science, University of KwaZulu-Natal, South Africa

## Abstract

An unpredicted outbreak of African animal trypanosomosis or nagana in 1990 in north-eastern KwaZulu-Natal necessitated an emergency control programme, utilising the extensive cattle-dipping system in the area, as well as a reassessment of the tsetse and trypanosomosis problem in the province. Since 1990, sporadic blood sampling of cattle at the dip tanks in the nagana-infested areas were undertaken to identify trypanosome species involved and to determine the infection prevalence in cattle. The distribution and species composition of the tsetse populations in the area were also investigated. From November 2005 to November 2007 selected dip tanks were surveyed for trypanosome infection prevalence. During April 2005 to August 2009 the distribution and abundance of tsetse populations were assessed with odour-baited H traps. The tsetse and trypanosome distribution maps were updated and potential correlations between tsetse apparent densities (ADs) and the prevalence of trypanosomosis were assessed. *Glossina brevipalpis* Newstead and *Glossina austeni* Newstead were recorded in locations where they have not previously been collected. No significant correlation between tsetse relative abundance and nagana prevalence was found, which indicated complex interactions between tsetse fly presence and disease prevalence. This was epitomised by data that indicated that despite large differences in the ADs of *G. austeni* and *G. brevipalpis*, trypanosome infection prevalence was similar in all three districts in the area. This study clearly indicated that both tsetse species play significant roles in trypanosome transmission and that it will be essential that any control strategy, which aims at sustainable management of the disease, should target both species.

## Introduction

The discovery that *Trypanosoma brucei* Plimmer & Bradford was the cause of African animal trypanosomosis (AAT), also known as nagana, can be dated back to the 1880s when it was recorded for the first time in north-eastern KwaZulu-Natal (formerly known as Zululand), South Africa (Bagnall 1993; Bruce 1895; Steverding 2008). In 1895, Sir David Bruce stated that game animals were the reservoir hosts of the causative trypanosome species and that these protozoan parasites were transmitted between their mammalian hosts by tsetse flies (Diptera: Glossinidae) (Bruce 1895). Four species of tsetse, that is, *Glossina morsitans morsitans* Westwood, *Glossina pallidipes* Austen, *Glossina brevipalpis* Newstead and *Glossina austeni* Newstead have been recorded in South Africa. *G. m. morsitans* was the only species that was encountered in the most northern parts of the country (this included the Kruger National Park and parts of the Limpopo and Mpumalanga provinces), bordering onto Zimbabwe in the north and Mozambique in the east (Fuller 1923). In 1897 after a large reduction of cattle and wildlife during the rinderpest epizootic of 1896–1897, *G. m. morsitans* completely disappeared from South Africa (Fuller 1923). The other three species of tsetse, that is, *G. pallidipes, G. brevipalpis* and *G. austeni,* remained present in the former Zululand in the north-eastern part of KwaZulu-Natal Province (Fuller 1923). *Glossina pallidipes* was the predominant species in north-eastern KwaZulu-Natal, and based on its abundance it was considered the most important vector of AAT at that time. *G. brevipalpis* and *G. austeni,* which were restricted to areas with mostly dense vegetation, were not considered as important vectors of AAT. The sheer abundance of *G. pallidipes* was illustrated by large numbers of flies being trapped on certain occasions, that is, in 1932, 2 million flies were collected within a month after deployment of 1000 Harris traps and nearly 8 million flies over that entire year (Harris 1932).

The presence of these large numbers of tsetse flies in north-eastern KwaZulu-Natal resulted in severe outbreaks of nagana between 1942 and 1946 (Du Toit 1954). A campaign that was largely reliant on newly developed synthetic insecticides such as DDT (1,1,1-trichloro-2,2-di (4-chlorophenyl)ethane) and HCH (hexachlorobenzene) was launched between 1945 and 1952 to eradicate *G. pallidipes*. These chemicals were extensively used as part of residual aerial spraying campaigns that were aimed at creating a zone free of *G. pallidipes* in Zululand (Du Toit 1954). Surveys in 1953 failed to detect any *G. pallidipes* and it was concluded that this species was eradicated from Zululand (Du Toit 1954). The programme was very successful because it integrated several control tactics (aerial spraying, trapping and bush clearing). Furthermore, it was sustainable as the entire *G. pallidipes* population was targeted and KwaZulu-Natal is today still free of this species. This was one of the first tsetse fly control programmes that was implemented following Area-Wide Integrated Pest Management (AW-IPM) principles (Klassen 2005; Vreysen, Robinson & Hendrichs 2007).

An additional benefit from this campaign was the apparent removal of *G. brevipalpis* from the Hluhluwe–iMfolozi Park (Du Toit 1954). In areas where *G. brevipalpis* and *G. austeni* were found in the absence of *G. pallidipes,* no control efforts were implemented against these species, and consequently, these two species remained present in Zululand (Kappmeier, Nevill & Bagnall 1998).

From 1955 onwards, only sporadic cases of nagana were recorded in Zululand (Bagnall 1993, 1994). However, in 1990, a severe outbreak of nagana once again occurred in north-eastern KwaZulu-Natal. Cattle mortalities during this outbreak were exacerbated by the co-occurrence of a severe drought (Emslie 2005; Kappmeier *et al*. 1998). Emergency control measures were implemented that relied on the extensive dipping network that was mainly used for tick control in KwaZulu-Natal (Kappmeier *et al*. 1998). As part of the control measures, the active ingredient of the acaricide dipping product was changed from amitraz to the pyrethroid cyhalothrin for a period of 2 years (Bagnall 1993). Whereas the pyrethroid cyhalothrin is very effective for controlling both ticks and tsetse flies (Bagnall 1993), the efficacy of amitraz is limited to ticks and is considered less effective against dipteran flies (Hall & Fischer 1984). The adopted cattle-dipping regime in combination with animal treatments using trypanocidal drugs brought the disease outbreak under control (Kappmeier *et al*. 1998).

The 1990 outbreak illustrated that nagana had remained a serious debilitating problem in KwaZulu-Natal, with mixed infections of *Trypanosoma congolense* Broden and *Trypanosoma vivax* Ziemann (Bagnall 1993). A survey to determine the prevalence of *Trypanosoma* species in 1994 indicated the highest trypanosomosis prevalence in the Ubombo district of KwaZulu-Natal, while the lowest prevalence was found to be in the Hlabisa district, which surrounds the Hluhlwe–iMfolozi Park (De Waal *et al*. 1998). Periodic screening of cattle in the tsetse-infested area indicated that *T. congolense* was the dominant species (Mamabolo *et al*. 2009; Motloang *et al*. 2012; Van den Bossche *et al*. 2006), *T. vivax* being present to a lesser extent (Mamabolo *et al*. 2009; Motloang *et al*. 2014). Recently *Trypanosoma theileri* (Laveran) and *T. brucei* were detected based on the alignment of 18S rRNA gene sequences which were comparable with trypanosome sequences available at the National Centre for Biotechnology Information (NCBI) (Taioe 2014). Two sub-genotypes of *Trypanosoma congolense*, the savannah and kilifi were identified with PCR (Mamabolo *et al*. 2009). Mixed infections of these two sub-genotypes were also observed (Gillingwater, Mamabolo & Majiwa 2010). These surveys showed that trypanosome infections in cattle at dip tanks close to the Hluhluwe–iMfolozi Park were higher than at dip tanks further away from this game reserve (Motloang *et al*. 2014; Ntantiso *et al*. 2014; Van den Bossche *et al*. 2006).

Long-term management of nagana by dipping and/or curative treatment of infected cattle with trypanocidal drugs is neither cost effective nor sustainable (Bagnall 1994; Shaw 2009), and it became evident that a long-term solution to the nagana problem in South Africa could only be attained if vector control was to be included. Initial surveys showed that *G. brevipalpis* and *G. austeni* were still the only species present in KwaZulu-Natal (Kappmeier Green 2002).

**FIGURE 1:**
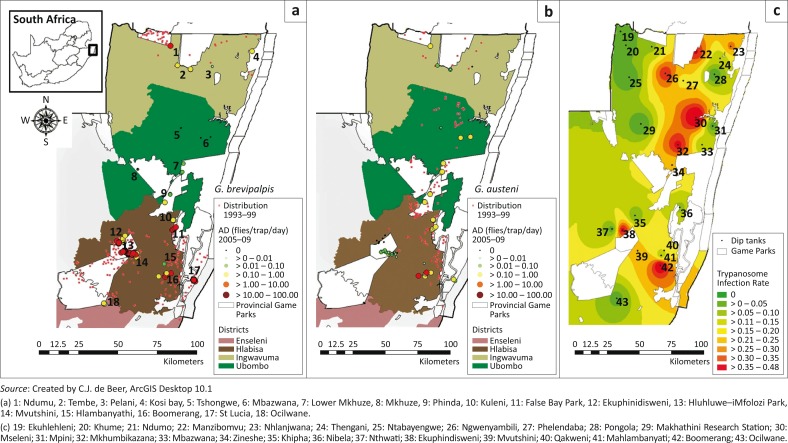
Apparent density of *Glossina brevipalpis* (a), *Glossina austeni* (b) and trypanosome prevalence in cattle at dip tanks (c) in north-eastern KwaZulu-Natal.

Between 1993 and 1999, extensive tsetse fly surveys were conducted in an area of approximately 12 000 km^2^ using odour-bated sticky XT traps (cross-shaped targets) to assess the distribution of each species (Figure 1a, b) (Kappmeier & Nevill 1999a, Kappmeier Green & Venter 2007). The survey indicated that *G. brevipalpis* was present in southern and northern bands, commonly associated with game reserves and other protected areas. The distribution of *G. austeni* was continuous from south to north and did not extend as far west as that of *G. brevipalpis*. *G. austeni* also was very common in communal farming areas (Kappmeier Green 2002). The 1993–1999 survey did not establish the southernmost distribution limit of *G. brevipalpis* and *G. austeni*, as the sampling frame was only designed to determine broad distribution limits. It was, however, suggested that the southernmost limit of the tsetse fly distribution in South Africa, and therefore Africa, could roughly be regarded as the southernmost extent of the Umfolozi River (Kappmeier Green 2002). The 1993–1999 survey also showed that *G. brevipalpis* and to a lesser extent *G. austeni* were present close to the coastal areas apparently not sampled in the 1950s (Du Toit 1954).

The entomological sampling data obtained from 1993 to 1999 (Kappmeier Green 2002) as well as environmental and climatic variables were used to develop a probability of presence model for these two species (Hendrickx 2002; Hendrickx *et al*. 2003). The model predicted a broader geographical distribution range for both *G. brevipalpis* and *G. austeni* than what was indicated by the survey data; however, Hendrickx *et al*. (2003) suggested that the model overestimated tsetse fly distribution.

Knowledge on the accurate distribution of the target insect, as well as the interaction of the insect with the causative agent it transmits, is vital for the successful implementation of any proposed control campaign and will directly affect its outcome, sustainability and cost (Shaw 2009; Vreysen *et al*. 2007). Additional ongoing efforts to generate continental as well as national Atlas of tsetse and AAT (Cecchi *et al*. 2014, 2015) will be benefited from continuously updated data sets.

The development of an improved trap for *G. brevipalpis* and *G. austeni* (Kappmeier 2000) and enhanced artificial odour system for *G. brevipalpis* (Kappmeier & Nevill 1999a) allowed a more effective sampling of both species in South Africa. Subsequently, as a starting point, the prediction model of Hendrickx (2002) was validated through selective trapping in the area.

From 2005 to 2009, researchers at the Agricultural Research Council-Onderstepoort Veterinary Institute (ARC-OVI) carried out various studies. These included a transect study, based on the results of the distribution survey and prediction model, to determine the distribution of *G. austeni* to the west of the Hluhluwe Dam (Esterhuizen, Kappmeier & Nevill unpublished data) and to assess fly presence and densities at dip tanks (Gillingwater *et al*. 2010; Mamabolo *et al*. 2009; Motloang *et al*. 2012; Ntantiso *et al*. 2014). In addition, tsetse population genetics (Koekemoer *et al*, unpublished data) and morphometric studies (De Beer *et al*. unpublished data) were conducted to assess the level of gene flow between the tsetse populations in KwaZulu-Natal and those in southern Mozambique and Swaziland.

The present paper collates the data of these different studies to update the existing maps on the distribution of tsetse flies and trypanosomosis in north-eastern KwaZulu-Natal. Correlation between tsetse apparent densities (ADs) and infection prevalence of the disease in livestock were also investigated.

## Materials and methods

### Study area

The tsetse-infested area (± 16 000 km^2^) in South Africa is confined to the north-eastern part of KwaZulu-Natal Province. The area stretches roughly from the Umfolozi River (-28.5204, 32.3123) in the south to the border of Mozambique (-26.8692, 32.8342) in the north, and from the Indian Ocean coast in the east up to the west of the Hluhluwe–iMfolozi Park (-28.33416, 31.691222) (Kappmeier Green 2002). Although some commercial cattle farms are present, it is predominantly a subsistence cattle-farming area with numerous communal farms that are interspersed with a number of protected areas.

The protected areas consist of provincial and private game parks and reserves. The area surrounding the extensive fresh water lake system, iSimangaliso Wetland Park, is a proclaimed world heritage site. These areas not only contain a wide variety of game animals, such as birds, rodents and smaller primates, but also large numbers of bigger mammals that are potential hosts for tsetse. The target area contains a number of state forests, mostly pine and eucalyptus plantations, and commercial sugarcane farms.

The climate is subtropical with average minimum temperatures ranging from 10.52 °C ± 1.26 °C in July (winter) to 21.71 °C ± 0.67 °C in January (summer). The average maximum temperature ranges from 24.65 °C ± 0.77 °C in July to 30.86 °C ± 1.38 °C in January. The average minimum relative humidity ranges from 39.92% ± 8.23% in July to 62.04% ± 6.39% in October, whereas the average maximum relative humidity ranges from 92.23% ± 3.73% in June to 94.94% ± 3.13% in February. The area receives an average maximum of 132.92 mm ± 92.95 mm of rain in January, and most of the precipitation is received in the hot season from October to March. However, it can also rain in the cold dry season from April to September with a minimum average 9.27 mm ± 9.68 mm of rain in July. On average, more rain is received in the coastal area as compared with the interior.

### Tsetse fly sampling

Tsetse fly collections were made at 18 sites located in four magisterial districts: Ingwavuma, Ubombo, Hlabisa and the northern part of Enseleni (Table 1; Figure 1a, b). A total of 77 odour-baited H traps (Kappmeier 2000; Kappmeier & Nevill 1999b) were deployed at these 18 sites from April 2005 to April 2009. The number of trap days ranged from 723 at Ocilwane (northern Enseleni) to 1661 at False Bay Park (eastern Hlabisa district). The traps were baited with artificial odour baits to enhance trapping of *G. brevipalpis* (Kappmeier & Nevill 1999a). These consisted of 1-octen-3-ol and 4-methylphenol at a ratio of 1:8 that were released at 4.4 mg/h and 7.6 mg/h, respectively. The chemicals were dispensed from seven heat-sealed sachets (7 cm ´ 9 cm) made from low-density polyethylene sleeves (wall thickness 150 µm) placed near the entrance of the trap. A 300 mL glass bottle that dispensed acetone through a 6-mm hole in the lid at a rate of *ca*. 350 mg/h was placed next to the trap (Esterhuizen 2007; Kappmeier Green 2002). Flies were collected in a 20% ethanol solution to which Savlon^®^ (Johnson & Johnson, Pharmedica Laboratories [Pty] Ltd. Rattray Road, East London, South Africa) (0.4 mL/L) and formalin (0.4 mL/L) had been added to preserve the sampled flies as well as to protect them from ant and spider predation. Traps were emptied and serviced every 14 days. The number of each species of tsetse fly collected over this period was counted, and results for each species expressed as AD, that is, the number of flies per trap per day.

**TABLE 1:** Tsetse H trap catches from April 2005 to August 2009 in the north-eastern KwaZulu-Natal.

Magisterial districts	Numbers on map	Tsetse sample site	Number of traps	Start date	End date	Total number of trap days†	*Glossina brevipalpis*		*Glossina austeni*	
	
							Collected	Apparent density	Collected	Apparent density
Ingwavuma	1	Ndumu	1	14-06-2006	26-08-2009	1169	6358	5.44	210	0.18
	2	Tembe	4	14-06-2006	26-08-2009	4606	1529	0.33	135	0.03
	3	Pelani	1	14-06-2006	26-08-2009	1169	11	0.01	0	0.00
	4	Kosi bay	2	19-04-2006	26-08-2009	2262	444	0.20	1	< 0.01
	Total/district	-	8	14-06-2006	26-08-2009	9206	8342	0.91	346	0.04
Ubombo	5	Tshongwe	2	04-07-2006	25-08-2009	2275	0	0.00	7	< 0.01
	6	Mbazwana	2	14-06-2006	11-08-2009	1750	0	0.00	263	0.15
	7	Lower Mkhuze	3	14-06-2006	25-08-2009	3504	204	0.06	2022	0.58
	8	Mkhuze	2	25-10-2006	21-08-2009	2062	1	< 0.01	23	0.01
	9	Phinda	4	04-07-2006	25-08-2009	4550	261	0.06	2284	0.50
	Total/district	-	13	14-06-2005	25-08-2009	14 141	466	0.03	4599	0.33
Hlabisa	10	Kuleni	3	01-01-2006	21-08-2009	3984	697	0.17	856	0.21
	11	False Bay Park	4	01-04-2005	18-08-2009	6400	9894	1.55	3849	0.60
	12	Ekuphinidisweni	6	01-05-2006	23-08-2009	5371	8529	1.59	0	0.00
	13	Hluhluwe–iMfolozi Park	15	01-11-2005	19-08-2009	11 201	120 313	10.74	277	0.02
	14	Mvutshini	12	01-05-2005	18-08-2009	15 239	32 451	2.13	65	< 0.01
	15	Hlambanyathi	1	01-03-2006	31-08-2009	1279	28	0.02	1	< 0.01
	16	Boomerang	6	01-04-2005	03-08-2009	8199	11 627	1.42	6424	0.78
	17	St Lucia	3	23-10-2006	21-08-2009	3099	22 470	7.25	680	0.22
	Total/district	-	50	01-04-2005	31-08-2009	54 772	206 009	3.76	12 152	0.22
Enseleni	18	Ocilwane	6	01-11-2007	24-08-2009	3882	1632	0.42	0	0.00
	Total/district	-	6	01-11-2007	24-08-2009	3882	1632	0.42	0	0.00

**Total/study area**	**-**	**-**	**77**	**01-04-2005**	**31-08-2009**	**82 001**	**216 449**	**2.64**	**17 097**	**0.21**

† Sum of collection days of all traps per site.

### Trypanosomosis survey

A large number of communal dip tanks, constructed, maintained and operated by the Provincial Department of Veterinary Services, are routinely used for tick control and are homogeneously distributed throughout the tsetse-infested area. The tick control policy, as dictated by the Department of Veterinary Services, consists of weekly dipping of cattle in the summer and fortnightly dipping during the winter. Cattle in the communal farming areas as well as on the commercial farm Boomerang roam around freely. The trypanosome infection prevalence in cattle was determined at 27 dip tanks from November 2005 to November 2007 (Figure 1c). All animals that congregated at a particular dip tank were considered as one herd as they grazed together and were managed using the same animal husbandry practice (Emslie 2005; Ntantiso *et al*. 2014). Herd size was very variable and ranged from 13 to 6411 animals, and the number of cattle owners at each dip dank was likewise very variable and ranged between 5 and 296. Cattle were screened on the day they were scheduled for routine dipping. These herds were divided into groups of 30 to 40 cattle, and 2 to 3 animals in each group were randomly sampled. Table 2 lists the number of cattle sampled at each dip tank.

**TABLE 2:** Trypanosome infection rates as determined by buffy coat examination of live trypanosomes collected from cattle at dip tanks from November 2005 to November 2007 in the north-eastern KwaZulu-Natal.

Magisterial districts	Numbers on map	Dip tank	Dip tank ample date	Distance from game park (km)	Buffy coat examination		Trypanosome infection prevalence

					Positive	Negative	
Ingwavuma	19	Ekuhlehleni	25-05-2007	11.6	1	49	0.02
	20	Khume	01-06-2007	9.4	0	30	0.00
	21	Nduno†	30-05-2007	3.4	4	39	0.09
	22	Manzibomvu†	05-09-2007	0.3	18	32	0.36
	23	Nhlanjwana†	31-05-2007	2.6	11	31	0.26
	24	Thengani	17-05-2007	2.1	4	35	0.10
	25	Ntabayengwe	29-05-2007	26.9	0	38	0.00
	26	Ngwenyambili†	14-07-2006	7.5	11	21	0.34
	27	Phelendaba†	15-05-2007	8.2	7	31	0.18
	28	Pongola†	06-08-2007	3.3	0	30	0.00
	Total/district	-	-	-	56	336	0.14
Ubombo	29	Makhathini	06-07-2007	20.2	0	32	0.00
	30	Mseleni†	05-08-2007	1.9	22	24	0.48
	31	Mpini	05-11-2007	2	1	41	0.02
	32	Mkhumbikazare†	05-10-2007	7	18	31	0.37
	33	Mbazwana†	05-07-2007	4.3	4	34	0.11
	34	Zineshe†	06-04-2007	2.2	9	33	0.21
	35	Nibela†	27-01-2007	3	3	30	0.09
	Total/district	-	-	-	57	225	0.20
Hlabisa	36	Khipha	23-11-2006	7.3	2	34	0.06
	37	Nthwati†	15-02-2006	6	1	38	0.03
	38	Ekuphindisweni†	25-10-2006	3	10	17	0.37
	39	Mvutshini†	21-05-2007	3	7	27	0.21
	40	Qakweni†	15-08-2006	7.4	8	83	0.09
	41	Mahlambanyati†	30-11-2005	9.6	5	45	0.10
	42	Boomerang†	18-08-2006	9.5	24	30	0.44
	Total/district	-	-	-	57	274	0.17
Enseleni	43	Ocilwane†	09-07-2007	4.3	1	28	0.03
	Total/district	-	-	-	1	28	0.03

**Total/study area**	**-**	**-**	**-**	**-**	**171**	**863**	**0.17**

† Dip tanks used in trypanosome infection tsetse regression analysis.

Blood was collected from the tail or jugular vein of adult animals using 10-mL vacutainer tubes containing the anticoagulant EDTA (BD Vacutainer^®^; BD, Plymouth, United Kingdom) (Ntantiso *et al*. 2014). The blood was transferred to micro-haematocrit centrifuge capillary tubes (Marienfeld-Superior, Lauda-Königshofen, Germany) that were sealed with Cristaseal (Hawksley) and centrifuged in a haematocrit centrifuge for 5 min at 9000 rpm. The buffy coat of each specimen was extruded onto a microscope slide, covered with a cover slip and examined for motile trypanosomes under a compound microscope using a 40-times magnification (Paris, Murray & McOdimba 1982). The trypanosomosis infection prevalence at each dip tank was expressed as the number of cattle that had a positive identification of trypanosomes in their buffy coat as a proportion of the number of cattle screened.

### Data analysis

All data were analysed using the statistical software GraphPad Instat (version 3.00, 2003). Tsetse relative abundance was expressed as the AD of each species, that is, the number of flies collected per trap per day. For comparison of the relative abundance of *G. brevipalpis* and *G. austeni* populations, a paired test was used to differentiate between mean tsetse fly AD. The data were not normally distributed and the non-parametric Wilcoxon matched-pairs test was used. To evaluate the AD of each tsetse species per district, a one-way analysis of variance (ANOVA) was used to differentiate between the mean tsetse fly AD. The data were not normally distributed, thus a non-parametric method (Kruskal–Wallis test) was used. Additionally, Dunn’s multiple comparison tests were used if the *p* value < 0.05. Proportional differences in trypanosome infection prevalence were determined with chi-square (*χ*^2^) analysis with the Yate’s continuity correction. Linear regression analysis was carried out on trypanosome infection prevalence and tsetse fly AD, as well as infection prevalence and distances from game reserves. All statistical tests were done at the 5% significance level.

Maps were developed in ArcGIS Desktop 10.1 (ESRI, 2011). For the trypanosome infection prevalence, an inverse distance weighted interpolation method was used with a power 2 function and a variable search radius setting at 10 points.

## Results

### Tsetse distribution and abundance

A total of 77 H traps were deployed at 18 sites located in four magisterial districts, that is, Ingwavuma, Ubombo, Hlabisa and Enseleni (Table 1; Figure 1a, b). These traps collected a total of 216 449 *G. brevipalpis* (AD = 2.64 flies/trap/day) and 17 097 *G. austeni* (AD = 0.21 flies/trap/day) between 01 April 2005 and 31 August 2009 (Table 1; Figure 1a, b).

While both tsetse species were collected in the three northerly districts, only *G. brevipalpis* was trapped (with six H traps) at Ocilwane in the northern part of the Enseleni district in the south (Table 1; Figure 1a). The overall AD of *G. brevipalpis* (2.64 flies/trap/day) was significantly higher (*p* < 0.001) than that of *G. austeni* (0.21 flies/trap/day). This might, however, be an artefact resulting from the intrinsic biases of the trapping system. Comparison of the ADs in the three northerly districts indicated that the AD of *G. brevipalpis* was significantly higher than that of *G. austeni* in the Ingwavuma (*p* = 0.008) and Hlabisa (*p* < 0.001) districts (Table 1; Figure 1a, b). The AD of *G. brevipalpis* (0.03 flies/trap/day) in the Ubombo district was significantly lower than that of *G. austeni* (0.33 flies/trap/day) (*p* < 0.002) (Table 1).

*Glossina brevipalpis* was most abundant in the Hlabisa district (3.76 flies/trap/day), and the AD was significantly higher (*p* < 0.001) than that in the Ubombo district (0.03 flies/trap/day); an area where previously no *G. brevipalpis* were collected (Kappmeier Green 2002) (Table 1; Figure 1a).

Significantly higher numbers (*p* = 0.025) of *G. austeni* (AD = 0.33 flies/trap/day) were collected in the Ubombo district as compared with the Hlabisa (0.02 flies/trap/day) and the Ingwavuma districts (0.04 flies/trap/day) (Table 1). No *G. austeni* was trapped at Ocilwane in the Enseleni district (Table 1; Figure 1b). These trapping data indicated that the Monzi forest in the Hlabisa district maintained the most southerly distribution of *G. austeni* in KwaZulu-Natal (and Africa).

The greatest variations in ADs among sites within the same district (Table 1) were observed in the Hlabisa district for both *G. brevipalpis* (*p* < 0.001) and *G. austeni* (*p* < 0.001). The AD of *G. brevipalpis* in the Hluhluwe–iMfolozi Park (10.74 flies/trap/day) was significantly higher than that observed at Kuleni (0.17 flies/trap/day). There were also significant differences in AD of the *G. brevipalpis* populations of St Lucia (7.25 flies/trap/day) and Kuleni (0.17 flies/trap/day). *G. austeni* was most abundant at Boomerang (0.78 flies/trap/day), and this AD was significantly higher than that obtained in the Hluhluwe–iMfolozi Park (0.02 flies/trap/day). In the Ubombo district, the AD of *G. brevipalpis* at False Bay Park (1.55 flies/trap/day) was significantly higher (*p* = 0 .044) than that at Phinda (0.06 flies/trap/day) and Lower Mkhuze (0.06 flies/trap/day). The AD of *G. austeni* was significantly higher at False Bay Park (0.60 flies/trap/day) (*p* = 0.002) in the Ubombo district as compared with the AD in Mkhuze (0.01 flies/trap/day). Finally, in the Ingwavuma district there were no significant differences in relative abundance between individual sites for both *G. brevipalpis* (*p* = 0.114) and *G. austeni* (*p* = 0.113).

### Trypanosome infection at dip tanks

A total of 1034 blood samples were collected from cattle at 25 dip tanks in the tsetse-infested area and were examined microscopically for the presence of trypanosomes (Table 2). The number of cattle screened per dip tank ranged from 27 at Ekuphindisweni to 54 at Boomerang (Table 2).

Data of trypanosome prevalence (Table 2) were, as was done for the tsetse data, grouped per magisterial districts (Table 1). The results showed that trypanosome infection was widespread in north-eastern KwaZulu-Natal with positive infected cattle found at 21 (84.0%) of the 25 dip tanks surveyed (Table 2; Figure 1c). Trypanosome prevalence ranged from 20% in the Ubombo district to 3% in the Enseleni district and was not significantly different (*p* = 0.05, *χ*^2^ = 7.91, *df* = 3) between the four magisterial districts. Notwithstanding the fact that there were no significant differences between the four districts, the proportional representation of positive animals at the 25 individual dip tanks differed significantly (*p* < 0.001, *χ*^2^ = 173.30, *df* = 24). Significant differences in the proportion of positive animals per dip tank were also found within the districts of Hlabisa (*p* < 0.001, *χ*^2^ = 51.462, *df* = 6), Ingwavuma (*p* < 0.001, *χ*^2^ = 59.078, *df* = 9) and Ubombo (*p* < 0.001, *χ*^2^ = 51.211, *df* = 6). These proportional differences in infection prevalence found between individual dip tanks seemed to be linked to the distance between the dip tank and a protected area or game reserve.

The highest trypanosome prevalence (48%) was recorded at the Mseleni dip tank which was situated next to the northern parts of iSimangaliso Wetland Park in the Ubombo district (Table 2). Similarly, trypanosome prevalence in cattle was high at dip tanks that were close to protected areas and/or game parks (Figure 1c). Especially in the Hlabisa district, a significant positive linear regression (*r*^2^ = 0.62, *p* = 0.04) was evident between trypanosome prevalence and the distance between the dip tank and the protected areas or game parks. The same trend was also observed for the other two districts, but it was not significant (Ingwavuma: *r*^2^ = 0.22, *p* = 0.17 and Ubombo: *r*^2^ = 0.19, *p* = 0.41). The correlation between infection prevalence and distance from the game parks was further epitomised by the absence of infected animals at the two dip tanks located more than 20 km further from a game park (Table 2).

### Tsetse relative abundance and trypanosome infection prevalence

The trypanosome prevalence in cattle, as determined with the buffy coat technique, at the 18 dip tanks was correlated with the tsetse relative abundance, as determined with odour-baited H traps. Only dip tanks that had H traps deployed in a 12 km radius were selected for the regression analyses (Table 2). No linear correlation could be found between trypanosome prevalence and the relative abundance of either *G. brevipalpis* (*r^2^* = 0.01, *p* = 0.68), *G. austeni* (*r^2^* = 0.05, *p* = 0.40) or the total tsetse catches (*r^2^* = 0.04, *p* = 0.43). However, at some of the dip tanks with high trypanosome prevalence in the cattle, for example, Mseleni (48%) and Mkhumbikazana (37%) in the Ubombo district, the AD for *G. austeni* was relatively high in the absence of *G. brevipalpis* (Figure 1a, b). In contrast, at the Ekuphindisweni dip tank in Hlabisa district where the screened cattle showed a similar high infection prevalence (37%), the relative abundance of *G. brevipalpis* was high, in the absence of *G. austeni* (Figure 1a, b). The relative abundance of both *G. brevipalpis* and *G. austeni* was relatively high at the dip tank on the commercial farm Boomerang that is located to the west of and next to the southern extent of the iSimangaliso Wetland Park and where the highest trypanosome prevalence (44%) was recorded in the Hlabisa district. These apparent discrepancies in AD of tsetse flies at the dip tanks with high trypanosome infection prevalence indicate that a number of factors, such as the tsetse and trypanosomosis monitoring regimes used (a trap placement radius of 12 km could be too large), may influence the outcome of such a regression.

## Ethical considerations

Materials used in the study posed no health risk to researchers, and no vertebrate animals were harmed. The study was done as part of a project on National Assets (000773) at the ARC-OVI in collaboration with the Food and Agriculture Organization (FAO)/International Atomic Energy Agency (IAEA) Division of Nuclear Techniques in Food and Agriculture and the Department of Technical Cooperation of the IAEA under project RAF 5069.

## Discussion

Baseline data on tsetse distribution, relative abundance and species composition, as well as the extent of the trypanosomosis problem, are essential for the development of an appropriate cost effective, sustainable area-wide control strategy (Leak, Ejigu & Vreysen 2008; Shaw 2009; Vreysen 2005).

Previous surveys, using XT sticky traps, indicated that the *G. brevipalpis* population was restricted to two distinct bands in the north and south of the tsetse fly infested area in KwaZulu-Natal (Kappmeier Green 2002) (Figure 1a). These two bands were also more or less defined in the tsetse distribution prediction model for this species (Hendrickx *et al*. 2003; Kappmeier Green, Potgieter & Vreysen 2007), except for a few patches predicted centrally. During the recent surveys, the H trap sampled low numbers of *G. brevipalpis* in at least five sites in a central area in the tsetse-infested belt and located east of the Mkuzi Game Reserve and north of the southern previously defined distribution band (Figure 1a). Therefore, these positive trap catches could in part be used to update the prediction model of Hendrickx (Hendrickx *et al*. 2003) and indicate that *G. brevipalpis* is also present in the central and southern parts of Ubombo district, where it was previously not sampled. Whether this distribution is patchy or continuous still needs to be verified.

During the 1993–1999 surveys (Kappmeier Green 2002), *G. austeni* was sampled at the Hluhluwe Dam situated east of Hluhluwe–iMfolozi Park (Figure 1b). A transect study was therefore carried out along the Hluhluwe River, starting at the Hluhluwe Dam and progressing to the west into Hluhluwe–iMfolozi Park, with the aim to determine if *G. austeni* could be found in the park as predicted by the distribution model of Hendrickx *et al*. (2003). All traps along the transect sampled *G. austeni*, but at very low ADs (0.02 flies/trap/day) (Table 1). The presence of *G. austeni* along this transect also confirms the accuracy of the *G. austeni* distribution prediction model (Hendrickx *et al*. 2003). *Glossina brevipalpis* was likewise trapped in the transect traps, but this was expected considering that it had previously been collected inside and outside the Hluhluwe–iMfolozi Park (Kappmeier Green 2002). Taken into account that *G. austeni* is primarily restricted to and does not disperse far from dense vegetation (Esterhuizen 2007; Esterhuizen *et al*. 2005), and in view that bush clearing in the communal farming areas has increased considerably (Kappmeier *et al*. 1998), it is likely that game reserves and protected areas will become more important in sustaining *G. austeni* populations in the future.

The survey data of 1993–1999 indicate the southernmost extent of the Umfolozi River (-28.5204, 32.3123) to be the most southerly distribution of *G. brevipalpis* (Kappmeier Green 2002). *Glossina austeni* was not trapped south of the St Lucia estuary, where the Umfolozi River enters the lake (Kappmeier Green 2002). The tsetse prediction model of Hendrickx *et al*. (2003) suggested a very low probability of occurrence for *G. austeni* in the south of the Umfolozi River, but a much higher probability of occurrence for *G. brevipalpis*. During the 2005–2009 surveys, *G. brevipalpis* was collected at the Ocilwane dip tank which is 5 km south of the Umfolozi River – an area which had not previously been surveyed (Figure 1a). The presence of *G. brevipalpis* at this site also corresponds to what was predicted by Hendrickx *et al*. (2003), so that the predictions were confirmed at this site. The results of the recent surveys also indicate that the most southern limit of *G. brevipalpis*, and the factors that will determine this in the area, remain undefined. Available data indicate that the southern limit for *G. austeni* seems to be defined as the Umfolozi River, as was previously assumed (Kappmeier Green 2002). Similarly, the most western extent of the distribution of each species needs to be verified. Available trap catches indicate that the most western distribution limit of *G. austeni* was -28.10357, 32.08377 (in Hluhluwe–iMfolozi Park), whereas *G. brevipalpis* was collected much further west at -28.04500, 32.03900, that is, west of Ocilwane (Figure 1a, b).

The trap catches have consequently been used to update the tsetse distribution prediction models for both *G. brevipalpis* and *G. austeni* (Hendrickx 2007). Since only data up to 2009 are presented, ongoing and future surveys will have to be processed and incorporated into these distribution maps and models. The available trap data have partly validated the prediction model, emphasising the fact that such models can be valuable tools for the planning of tsetse and trypanosomosis intervention practices and their value should not be underestimated. Furthermore, the new distribution data will need to be taken into consideration in the suggested tsetse control strategy proposed by Kappmeier Green *et al*. (2007), which needs to be modified accordingly.

Trypanosome infections in cattle were recorded at 21 of the 25 dip tanks which clearly showed that the disease was still abundant and highly prevalent in north-eastern KwaZulu-Natal at the time of the trypanosomosis survey (Figure 1c). A potential shortcoming of the trypanosome survey may have been that infections were not identified to species level. It was, however, previously indicated that *T. congolense* is the dominant species in the area (Mamabolo *et al*. 2009; Motloang *et al*. 2012; Van den Bossche *et al*. 2006).

The data on the spatial distribution of the trypanosome infection in north-eastern KwaZulu-Natal indicated that the disease was widespread in cattle, that is, from areas close to the Mozambique border in the north to a dip tank close to the Umfolozi River in the south, covering the entire length of the tsetse-infested area of north-eastern KwaZulu-Natal (Figure 1c). In general, higher infection rates were found in dip tanks located near game reserves and protected natural areas (Figure 1c), an observation also made by Van den Bossche *et al*. (2006) and Ntantiso *et al*. (2014) for dip tanks near the Hluhluwe–iMfolozi Park. This game–livestock–tsetse interface poses a high risk for cattle getting infected not only for the Hluhluwe–iMfolozi Park (Ntantiso *et al*. 2014), but possibly throughout north-eastern KwaZulu-Natal.

In 1994, the highest prevalence of trypanosomosis was recorded in the Ubombo district (Kappmeier *et al*. 1998). Although in the present study the dip tank with most of the cattle infected (Mseleni 48%) was found in this district, the trypanosome prevalence was not significantly different from that obtained in the Ingwavuma and Hlabisa districts confirming that trypanosomosis remains a problem throughout the whole area.

The absence of a significant linear correlation between trypanosome prevalence and the relative abundance of tsetse flies in north-eastern KwaZulu-Natal may partly be attributed to the coexistence of the two tsetse species, each with a different vectorial capacity and/or competence. Previous studies indicated that the vector competence of *G. austeni* for *T. congolense,* the most abundant *Trypanosoma* species in north-eastern KwaZulu-Natal, was significantly higher than that of *G. brevipalpis* (Motloang *et al*. 2012). Our data seem to corroborate this greater vector competence of *G. austeni* in view of the relative high trypanosome prevalence recorded in cattle at the Mseleni dip tank where only *G. austeni* flies were sampled (Mbazwana AD = 0.15 flies/trap/day). However, cattle at the Ekuphindisweni dip tank showed a similar high trypanosome prevalence, but *G. austeni* was not sampled in the traps; only *G. brevipalpis* was trapped in relatively high numbers (Ekuphindisweni AD = 1.59 flies/trap/day) indicating that *G. brevipalpis* was most likely responsible for transmission in this area. In general, high ADs of *G. brevipalpis* in the vicinity of dip tanks resulted in trypanosome infection rates that were as high as those found at dip tanks where *G. austeni* was present but at low ADs. Hendrickx *et al*. (2003) also noted that low *G. austeni* densities coincide with areas of higher trypanosomosis prevalence in the northern communal areas. However, the very high ADs of *G. brevipalpis* (on average 12.6 times higher than that of *G. austeni*) and the much lower trap catches of *G. austeni* might be an artefact resulting from the intrinsic biases of the trapping system. It is known that *G. austeni* responds poorly to traps (Kappmeier 2000), and it is not attracted to any of the artificial odours (Kappmeier & Nevill 1999a; Kappmeier Green 2002) used to bait traps. Essentially, mark–release–recapture studies will be needed to confirm the low efficacy of the sampling tool used for *G. austeni*.

If the relative abundance of G. breviplapis is indeed a reflection of actual population densities, it can be hypothesised that the high trypanosome infection prevalence in certain areas might be the result of the greater densities of *G. brevipalpis* despite their lower vector competence. The relative low vector competence of *G. brevipalpis* previously found in South Africa has probably resulted in an underestimation of the importance of *G. brevipalpis* in the epidemiology of nagana in north-eastern KwaZulu-Natal. Vector competence studies in the laboratory have shown that the susceptibility of *G. brevipalpis* for *T. congolensis* can be as high as 12.3% (Moloo, Okumu & Kuria 1998). In Uganda, it was shown that the infection rates for *T. congolensis* in *G. brevipalpis* collected in field could be 2.6% (Harley 1967a, 1967b; Moloo, Kutuza & Boreham 1980). In addition, trypanosome infection rates in tsetse flies are not constant and can change over time and between populations. In Uganda, infection rates varied considerably with the different seasons and with the age of the *G. brevipalpis* population (Harley 1966, 1967a, 1967b).

Both *G. brevipalpis* and *G. austeni* will readily feed on cattle (Clausen *et al*. 1998; Moloo 1993) and can therefore play a role in the transmission of trypanosomosis in KwaZulu-Natal. Similar average trypanosome infection rates in the three northerly districts seem to support this hypothesis even though the relative abundance and distribution of *G. austeni* and *G. brevipalpis* were clearly different.

## Conclusion

The dynamics of tsetse populations can fluctuate in space and time and need to be monitored regularly for the development and implementation of an effective control strategy. Differences in fly density, distribution and ecology imply that different control strategies may be needed for the control of both these species in the nagana-infected area. The role that game parks and other protected areas may play in sustaining tsetse populations, as well as the circulation of trypanosomes in game animals, and the potential dispersal of both the vector and pathogen from these areas need further investigation. Furthermore, it will be essential that any strategy that aims at finding a sustainable solution to the nagana problem in north-eastern KwaZulu-Natal needs to target both tsetse fly species.
